# Immediate Response to Chemotherapy in an Adult Neuroblastoma Patient Presenting with Cord Compression

**DOI:** 10.1155/2020/6401497

**Published:** 2020-02-19

**Authors:** Nedal Bukhari, Bachar Harfouch, Majid Shallal Alotaibi, Hulayel Al-Harbi, Omar Chamdine

**Affiliations:** ^1^Department of Medical Oncology, King Fahad Specialist Hospital, Dammam, Saudi Arabia; ^2^Department of Internal Medicine, King Fahd University Hospital, Imam Abdulrahamn Bin Faisal University, Dammam, Saudi Arabia; ^3^Department of Neurosurgery, King Fahad Specialist Hospital, Dammam, Saudi Arabia; ^4^Department of Neurology, King Fahad Specialist Hospital, Dammam, Saudi Arabia; ^5^Department of Pediatric Hematology Oncology and Stem Cell Transplantation, King Fahad Specialist Hospital, Dammam, Saudi Arabia

## Abstract

We report a case of a 31-year-old female patient with high-risk neuroblastoma (NBL) who presented with a history of static back pain and bilateral lower limb weakness for almost a month. Her primary tumor was located in the right paraspinal region, causing spinal cord compression (SCC). Chemotherapy was administered with an immediate clinical improvement noted after 24 hours of starting treatment. We herein report the efficacy of chemotherapy in an adult neuroblastoma (aNBL) patient presenting with spinal cord compression.

## 1. Introduction

NBL is the most common extracranial solid tumor in childhood that originates from the neural crest. It is almost exclusively a disease of children. It affects them in early childhood (>90% of cases diagnosed before 5 years of age) and is rare among adolescents and adults [1].

Neuroblastomas can invade the spinal canal causing SCC. Our patient had symptoms and signs of epidural spinal cord compression (ESCC) and thus received chemotherapy [2, 3].

In this case, we describe the response to chemotherapy manifested by a marked improvement in the neurological symptoms within a very short period of time.

## 2. Case Report

A 31-year-old female, previously healthy, referred from a community hospital with a 4-week history of back pain, associated with bilateral lower limb weakness. No symptoms of paresthesia, urinary retention, or constipation reported. She was on dexamethasone 16 mg/day for 5 days prior to transfer to our hospital with no noticeable clinical improvement.

The patient was unable to stand on the first encounter. Positive findings on neurological examination revealed a muscle power of 2/5 on hip flexion, hip extension, knee flexion, foot dorsiflexion, and plantar flexion bilaterally. Hyperreflexia noted in lower extremities.

An ultrasound-guided biopsy was done at the referring hospital. Pathology was reviewed at our institute, and diagnosis of neuroblastoma was confirmed. MYCN amplification was present indicating unfavorable histology.

**Figure 1:**
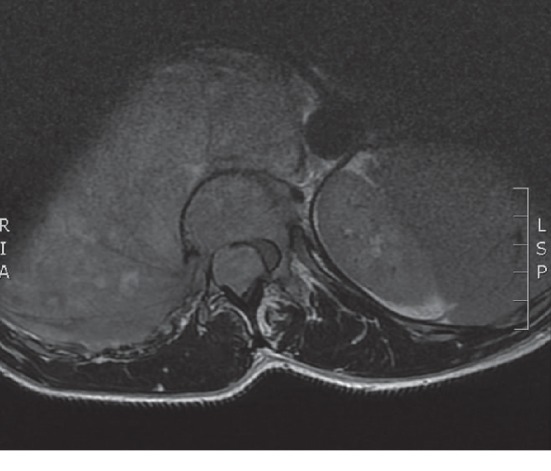
Severe compression of the cord with the absence of CSF around it.

Upon arrival of the patient, magnetic resonance imaging (MRI) of the spine was done and indicated severe SCC at the 12^th^ thoracic vertebra (T12) level (Figure 1).

A positron emission tomography (PET) scan was also done and revealed an 8 × 13 × 15 cm, right FDG-avid suprarenal mass with an SUV uptake of 6.7.

Bone marrow biopsy was also performed and was remarkable for invasion by neuroblastoma cells.

The patient's case was discussed in a multidisciplinary fashion, and she started treatment as high-risk NBL with induction chemotherapy. The first cycle of induction chemotherapy consisted of vincristine, doxorubicin, and cyclophosphamide as per the Children's Oncology Group (COG) protocol A3973. The same dose of dexamethasone at 16 mg/day was continued.

She started noticing an improvement in her back pain 24 hours after administering chemotherapy. Neurological examination 72 hours after the first cycle of treatment showed a muscle power of 3/5 on hip flexion, hip extension, knee flexion, foot dorsiflexion, and plantar flexion bilaterally. We evaluated the patient one week after induction, and she was able to stand up and walk with assistance. The plan was to proceed as per the COG A3973 protocol which comprises vincristine, doxorubicin, and cyclophosphamide alternating with cisplatin and etoposide.

The patient continued chemotherapy treatments with further symptomatic improvement.

**Figure 2:**
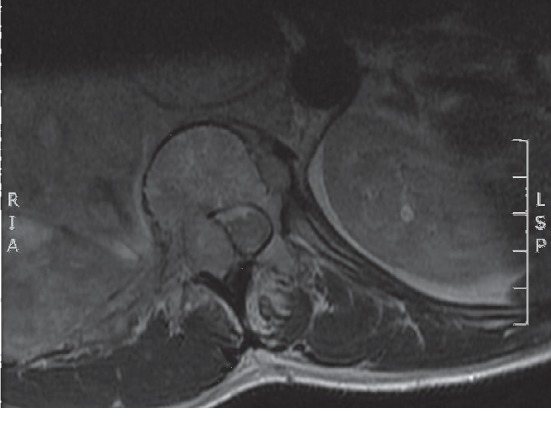
Image after the first cycle of chemotherapy indicating improvement in the compression with CSF present on the anterior aspect of the spinal cord.

**Figure 3:**
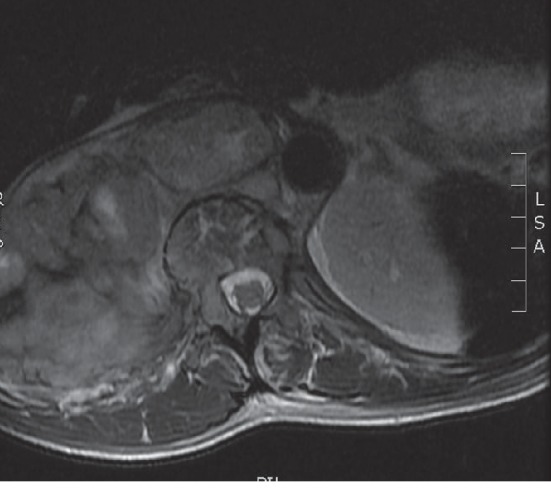
Complete decompression of the spinal cord with CSF surrounding it after 3 cycles of chemotherapy.

MRI thoracic spine was repeated after the first and third cycle of chemotherapy showed significant decompression of the spinal cord (Figures 2 and 3).

However, few days after cycle 6 of chemotherapy, she developed septic shock and unfortunately passed away shortly afterwards. Axial T2 cuts of thoracic spine MRI at T12 level taken 3 mm below the superior endplate of the vertebra.

## 3. Discussion

The term neuroblastoma (NBL) is used to refer to a spectrum of neuroblastic tumors that arise from the primitive sympathetic ganglion cells [1].

It is mainly a disease of the pediatric age group and is extremely uncommon in adults [3]. Due to the rarity of the adult NBL (aNBL), 1/10 million vs. 1/100,000 in children [4], the staging, risk assessment, and management in this age group are usually extrapolated from the pediatric literature [4]. In terms of biology, aNBL appears to be different from the pediatric counterpart. In one series of 44 adult patients, somatic mutations in *ATRX* and *ALK* were found in 58% and 42% of aNBL, respectively. Unlike our case, none of the patients harbored MYCN amplification. Treatment of high-risk neuroblastoma in the pediatric age group is intense and includes an induction chemotherapy, surgery, high-dose chemotherapy with autologous stem cell rescue, followed by radiation and biotherapy therapy with isotretinoin. More recently, targeted immune therapy with anti-GD2 monoclonal antibodies has become a standard of care in Europe and North America [5, 6]. The treatment of low and intermediate risk neuroblastomas is less intensive, and outcomes are typically better [6].

Spinal cord compression occurs approximately in 5–10% of newly diagnosed NBL patients, predominantly at the thoracic level [7]. Presenting symptoms can vary from loss of bowel and bladder control to complete paraplegia. This constitutes a neuro-oncological emergency and mandates urgent management to prevent irreversible long term deficits, especially if symptoms are of <4 weeks duration [8, 9]. The approach to such cases is debatable among physicians and varies from an institution to another. Available therapeutic options include surgery (decompressive laminectomy), chemotherapy, as well as radiation therapy. There is no clear evidence supporting the superiority of one modality over the other in terms of efficacy as evidenced by most studies [7, 9, 10]. We found two reported cases of adult patients with cord compression at presentation. Similar to our case, both patients underwent spinal cord decompression at diagnosis [11–14]. In general, it is advisable to limit surgery to patients who are progressing rapidly (within 24–72 hours), or if chemotherapy is not available, and in patients with low-grade tumors. Laminectomy and radiation therapy can be associated with a higher rate of spinal instability on the long term [10]. The use of glucocorticoids, after exclusion of lymphoma, may play a role in the improvement of early symptoms; however, it has not been shown to prevent late residual impairment [13]. In brief, treatment should be carefully tailored, taking into consideration the risk profile of the patient, as well as the factors mentioned above.

## Additional Points

The case demonstrates the effectiveness of chemotherapy in treating adult neuroblastoma patients presenting with spinal cord compression.
